# Covering the last mile for vaccination: Feasibility and acceptability of traditional birth attendant-based referral system in hard-to-reach areas in rural Pakistan

**DOI:** 10.7189/jogh.10.021303

**Published:** 2020-12

**Authors:** Ambreen Sahito, Siraj Ahmed, Zafar Fatmi

**Affiliations:** 1 Department of Community Medicine, Isra University Hyderabad, Pakistan; 2 Department of Health, Government of Sindh, Pakistan; 3 Department of Community Health Sciences, Aga Khan University, Karachi, Pakistan

## Abstract

**Background:**

Pakistan has a decent network of community-based workers including lady health workers (LHWs) and vaccinators. However, a major section of the population is not covered by LHWs/vaccinators, labeled here as hard-to-reach (HTR) areas, where immunization coverage is also considerably low. This study explored the feasibility of engagement of traditional birth attendants (TBAs) to improve EPI vaccination coverage in HTR areas in rural Sindh, Pakistan.

**Methods:**

This implementation research was conducted in two sub-districts of Sukkur (a district in Sindh Province). In an HTR selected intervention arm, TBAs were trained for vaccination and monetary incentives were provided to counsel and refer mothers for vaccination. While LHWs covered areas in the adjacent sub-district were provided with refresher training for vaccination only without any monetary incentive, and were considered as control arm. Considering the inherent differences in intervention and comparison group (HTR intervention area being worse regarding infrastructure and access), between groups and within group change in knowledge of TBA/LHWs and vaccination coverage was assessed before and after the intervention. Furthermore, focus group discussions were conducted with vaccinators, TBAs and LHWs and in-depth interviews with supervisors of vaccinators.

**Results:**

TBAs and LHWs’ vaccine related knowledge increased significantly after training (pretest vs post test score: 10.5 to 15.4). The BCG coverage improved 74.1% (percentage change) in TBA arm. While completion of vaccination (ie, Penta-3 coverage) increased by 147% from baseline following the intervention. The TBAs, LHWs, vaccinators and their supervisors all welcomed the initiative and considered it as a feasible option.

**Conclusions:**

Involvement of TBAs’ to form a referral system has potential to improve vaccine coverage and completion in HTR areas in Pakistan. The system is acceptable to the population and implementation is feasible due to availability of TBAs. However, in order to sustain the initiative minimal incentive need to be provided to TBAs to improve the vaccination coverage. Compared to establishing the infrastructure in HTR the intervention seems less costly however, it requires formal cost-effective or cost-benefit analysis.

Vaccination is among the most cost-effective public health interventions to prevent communicable diseases and reduce child mortality. Despite its proven value, vaccination coverage remains sub-optimal in many low- and middle-income countries (LMICs). The Expanded Programme on Immunization (EPI) was launched in Pakistan in 1978, but since the last decade, vaccination coverage has been stagnant [1]. Although reported coverage differs across areas, overall vaccination coverage remains low [2-4]. Recently reported complete vaccination coverage was only 54% in Pakistan. In addition to low enrolment of new-born in the immunization programme (for BCG), high dropout (or low completion rate) is an immense challenge [3]. Furthermore, inequities about vaccination coverage exist among provinces, urban vs rural residences, socioeconomic groups, and also related to remoteness of the area [1,3,5].

Factors contributing to low immunization coverage include lack of knowledge and ignorance among population leading to low demand for vaccines [1,6]. Demand side barriers can be tackled by creating awareness and appropriate counselling to actively involve parents in immunization [5,7,8]. Vaccinators and vaccination supervisors *(tehsil/taluka* supervisor vaccine – TSV, and district supervisor vaccine – DSV) constitute the main workforce of EPI in Pakistan. Chronic shortages and inequitable distribution of vaccinators, along with limited outreach activities due to logistic problems lead to poor vaccination coverage, particularly for inaccessible rural areas [9-11].

Engagement of community health workers (CHWs) has been shown to increase immunization coverage in LMICs [12,13]. Pakistan has fairly opulent network of community-based lady health workers (LHWs) who provide basic preventive, maternal and child health services. LHWs primarily work as social mobilizers for vaccination. In addition, they participate in EPI campaigns against polio, measles and tetanus. Immunization coverage is comparatively better in areas served by LHWs [14,15]. However, LHWs merely cover 55% of the population [14,15]. In the remaining 45% of LHW-unserved areas, which are considered here as hard-to-reach (HTR) rural remote areas, formal health care and vaccinators are not available. In HTR, maternal and child health care is mainly provided by traditional birth attendants (TBAs). About 34% of births overall and 40% in rural areas in Pakistan takes place outside health facilities, mainly assisted by TBAs at home [16]. Besides assisting in childbirth, many TBAs are hired by mothers to help in their postnatal period even if they have delivered the baby at a health facility. Therefore, TBAs are often the first-level care providers for the new-borns. Despite potential benefits, there have been limited attempts to systematically involve TBAs to improve coverage for routine immunization. Though during polio campaigns TBAs are also engaged when extraordinary arrangements and management efforts are made to provide coverage in HTR areas. Considering this, this study explored the feasibility of engagement of TBAs to improve EPI vaccination coverage in HTR areas in rural Sindh, Pakistan.

## METHODS

The study was conducted in a rural district (Sukkur) in Sindh province of Pakistan. The district spreads over 5165 sq. km and is divided into four *talukas* (sub-districts). Among them two sub-districts, Salehpat and Rohri, were selected as TBAs and LHWs arm, respectively, for implementing the study. The two sub-districts had inherent systematic differences between them in accessibility and health infrastructure, Rohri being more accessible and had better infrastructure than Salehpat (**Table 1**). The Salehpat and Rohri were purposely selected in consultation with the district health administration because vaccination coverage was among the lowest in the district in these areas. Salehpat is very remote with little public health infrastructure and few available health workers, while Rohri is comparatively better in infrastructure with fair availability of health care staff belonging to public sector [17].

**Table 1:** Characteristics of study arms (areas)

	Rohri (LHW arm)	Salehpat (TBA arm)
Geography	Riverine or kacha area	Mostly desert spread over 80 km
Administrative units	11 UCs and 67 dehs	3 union councils and 87 dehs
Health facilities	16 health facilities and EPI centres	4 health facilities and EPI centres
Vaccination-related human resources	39 vaccinators and 239 LHWs	8 vaccinators and 7 LHWs

LHW – lady health workers, TBA – traditional birth attendants

A mixed method study design was employed: a pre and post intervention design (quasi-experimental) with a control arm coupled with a qualitative inquiry. The aim of the quasi experimental study was to assess the improvement in the new-born enrolment rate for BCG and vaccine completion (Penta-3) and reduction in drop-out rate for vaccination by engaging TBAs.

### Intervention steps

**Figure 1:**
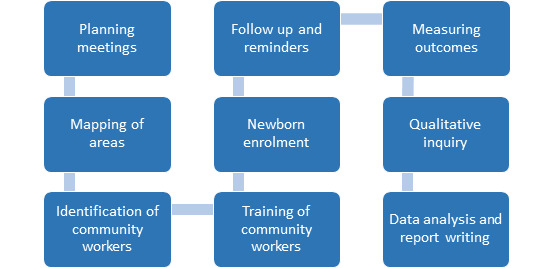
Flow diagram of project activities: Intervention processes and steps.

**Figure 2:**
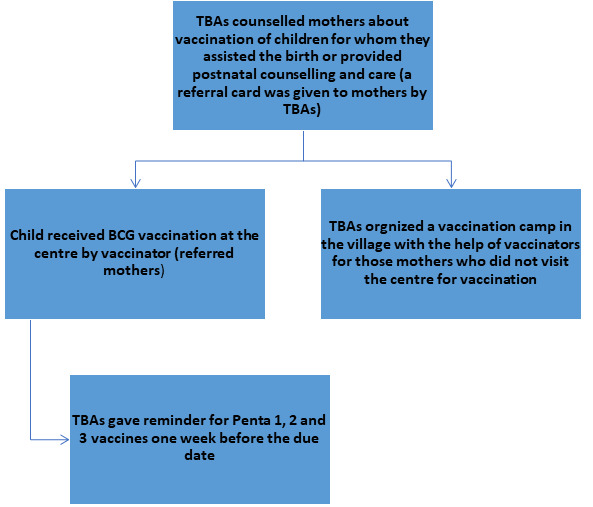
Activities of traditional birth attendants (TBAs) and vaccinators in the TBA arm, Salehpat.
- TBAs were given an incentive of PKR1000 (~US$8.0 at conversion rate of PKR128 = US$1 at the time of study), for achievement of each complete child vaccination. The complete vaccination of a child was defined when they received BCG, polio at birth and three doses of polio and Penta-3 vaccines. Measles vaccination were not considered to record completion due to short duration of the project. The TBAs were involved in counselling, referring and ensuring the timeliness of vaccination.

The following steps were undertaken to carry out the intervention (**Figure 1**):

- First, the strategic planning meetings were conducted with the district EPI staff at all levels – vaccinators, TSVs, DSVs, district focal persons for vaccine, additional deputy commissioners (ADCs), and district health officers (DHOs).
- Initial visits and the scoping exercises were conducted in consultation with the health administration to identify HTR areas in the district. Two union councils (UC), Laljurio Shabani in Salehpat and UC Ali Wahan in Rohri, were purposely selected after discourse. UC Laljurio Shabani had a total population of 40,380 and vaccination was provided by two vaccinators and three LHWs. Considering paucity of formal healthcare workers, TBAs were trained in this UC. UC Ali Wahan had a population of 25 526, with relatively improved access and infrastructure with 12 LHWs and one vaccinator. This UC served as the control (LHW) arm for the study.
- A joint meeting between TBAs and vaccinators was organized for establishing formal contact between them. The liaison between LHWs and vaccinators was old and was already well recognized and established in a formal way. In order to distinguish that the referral of child was made by a CHW who belonged to the intervention arm, a card was given to TBAs in addition to a registration sheet for keeping the records.
- TBAs and LHWs were trained for enrolment and referral of children for vaccination (**Figure 2**). They were also educated about vaccination, its importance, EPI schedule, common misconceptions and how to counsel parents (documents available with government health department). Several TBAs were escorted by their male family member so they were also made part of training, particularly to keep the records of referral of the child and vaccination.

### Sample size

The sample size was calculated using NCSS Pass version 8 software. Keeping 80% power and 5% level of significance with at least 10% expected reduction in the dropout; a sample of 180 participants in the intervention arm and 400 participants in the control arm were needed. Furthermore, keeping 5% non-response and lost to follow-up, the minimum sample was inflated to 190 in the intervention arm and 420 in the control arm.

We also did a comparison of the intervention arm with overall district coverage, thus there was a much larger sample for the control arm.

### Data collection and analysis

A questionnaire was used to assess TBAs and LHWs vaccine-related knowledge (their components described above) before and after the training using paired *t* test. The data from the administrative reports of the district EPI office was used for estimating the change in vaccination (BCG, Penta-3 and dropout) coverage. Dropout was calculated by subtracting Penta-3 coverage from BCG coverage. Vaccination coverage was compared between the TBA and LHW arms. We calculated both absolute and percentage change in coverage between pre- and post-intervention periods, allowing comparison for between-group and within-group differences. Besides, we compared the overall vaccination coverage (for vaccination coverage trend) during the intervention in whole district (Sukkur) to adjust for any extraneous factors which may have influenced the vaccination coverage in the entire district during the project period.

Data accuracy, consistency and completeness were ensured by cross-checking of a sub-sample of children from the intervention and control arms. The cross-check was done by investigators themselves by visiting the households and reviewing of vaccination records.

Data were analysed with SPSS version 23 (IBM corporation, Armonk NY, USA). Pre- and post-intervention knowledge levels (scores) were analysed using paired *t* test, and χ^2^ was used to compare percentage change in vaccination between groups and within arms before and after the intervention.

### Qualitative study

The aims of the qualitative inquiry were to: determine the experience of TBAs and LHWs regarding intervention, and determine the acceptance of TBAs by formal health care providers and perceived barriers of involvement of TBAs in vaccination.

Six in-depth interviews (IDIs) were conducted with the district focal person vaccine, DSV, TSV, vaccinators of study area (sub-districts) and National Stop Transmission of Polio (NSTOP) officer (in charge of vaccination who works with district administration and appointed by Centre for Disease Control US in each district). Furthermore, two focus group discussions (FGDs) were carried out with LHWs and TBAs who were involved in the project. A few parents (n = 12) were also interviewed during visits to households. IDIs and FGDs were conducted using semi-structured interview guidelines. Data was translated and transcribed for thematic content analysis and narrative summaries were provided.

The ethical review board of Health Services Academy Islamabad provided ethical approval. Informed written consent was signed by study participants (thumb impressions were taken after reading aloud the consent form for illiterate participants). Participant’s identification details were kept confidential.

## RESULTS

### Description of programme areas, TBAs, LHWs and population

A total of 12 LHWs were trained, covering about 60% population in the selected LHW arm UC (Ali Wahan). All LHWs had formal education, age ranged between 22 and 45 years. While a total of 23 TBAs were trained, covering 30% of the population in the selected UC (Laljurio Shabani). TBAs had no formal education, and their age ranged between 35-70 years. They had experience of 4-30 years in assisting births, which they gained informally through family members (mother, grandmother, aunty, sister). On average TBAs were assisting 3-5 births in a month. TBAs do not charge fixed fees for services but everybody pays them what they can afford. They usually do not receive cash but were given presents like scarf, clothes or sometimes cash US$2.5-4 (PKR300-500 at conversion rate of PKR128 = US$1 at the time of study). Three TBAs were also working in polio campaigns as volunteers with a moderate stipend.

### Change in knowledge about vaccination

**Figure 3:**
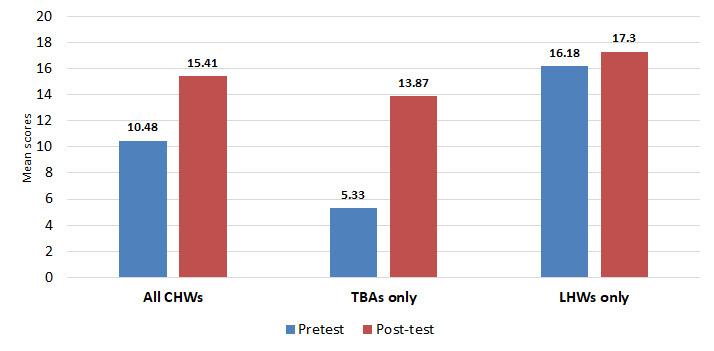
Change in vaccine-related knowledge (comparison of mean scores) among traditional birth attendants (TBAs) and lady health workers (LHWs) arms and combined TBA/LHWs as Community Health Workers (CHWs) before and after intervention.

Vaccine-related knowledge of TBAs and LHWs increased significantly after training (*P* < 0.01). The overall increase in knowledge was higher for TBAs, who had a 250% increase in score from the baseline (**Figure 3**).

### Vaccination coverage in programme areas

In the LHW arm, 56 children were enrolled in five months for BCG. While in the TBA arm, 240 children were enrolled in only 40 days. Among these 240 children, 171 were vaccinated at the appropriate age (within one week of date of EPI schedule). The project team requested TBAs to stop the enrolment after enrolling the required sample size, as there were limited funds available to incentivize them and continue with the program.

**Figure 4:**
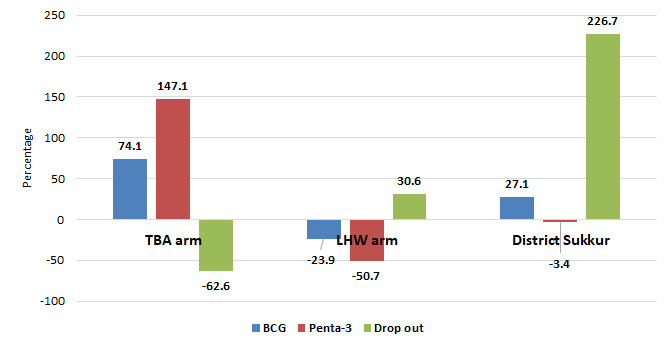
Percentage change in vaccination coverage from the baseline in traditional birth attendants (TBAs) and lady health workers (LHWs) and overall in Sukkur district.

As compared to baseline, an overall increase was observed in BCG coverage in both TBA arm and the district. However, the highest increase from the baseline (74% BCG and 147% Penta-3) was observed in the TBA arm. Importantly, there was an overall 226% increase in vaccine dropout in the district overall. However, in the TBA area, dropout decreased by 62% from the baseline during the same period (**Figure 4**).

**Table 2** shows the vaccine coverage trend in both the TBA and LHW arms, and overall vaccine coverage in Sukkur district during 2016 and 2017. In 2017, overall, there was increase in BCG in both UCs (36% in LHW and 37% in TBA arm) and overall district (4%) from 2016. Penta-3 coverage in 2017 also increased in both arms in 2017, but it was substantially higher in the TBA arm (146% increase from baseline) compared to LHW arm (32%) from 2016.

**Table 2:** Difference in vaccination coverage before and after the intervention in selected Union Councils (UC)

	LHW arm (UC Ali Wahan)			TBA arm ( UC Lal Jurio)			Sukkur District		
	**BCG**	**Penta-3**	**Dropout**	**BCG**	**Penta-3**	**Dropout**	**BCG**	**Penta-3**	**Dropout**
2016	70%	41%	29%	59%	26%	33%	110%	73%	37%
2017	95%	54%	41%	81%	64%	17%	114%	83%	31%
Difference	25%	13%	12%	22%	38%	-16%	4%	10%	-5%
% difference	36	32	41	37	146	-48	4	13	-13

LHW – lady health workers, TBA – traditional birth attendants

### Findings from qualitative inquiry

#### Perceived effectiveness of the intervention by stakeholders

During the FGDs with TBAs, they considered training to be useful in increasing their knowledge of vaccines and they felt empowered in counselling parents and referring children for the vaccination. TBAs believed parents have become aware and responsible. Initially, TBAs had to work hard to motivate parents but now parents’ attitudes have improved and they were coming to TBAs for vaccination. One of TBA informed:

“We had to explain importance of immunization and counsel parents initially now people have become aware. They understand the importance of immunization, and they reach to us for vaccination.”

Parents (n = 12) in remote villages considered TBAs as very useful for vaccinating children. Since this was the first time their children ever received vaccines, parents wanted TBAs to continue their activity. As one household said:

“Its first time our children are being immunized due to efforts of TBAs. Our sisters (TBAs) are coming to our doorsteps and arrange vaccination camps in our villages.”

All vaccinators agreed that TBAs were very helpful in improving vaccination coverage and follow-up for children. All EPI managers (at all levels) welcomed the initiative of TBAs involvement in routine vaccination. They claimed that the coverage achieved in the TBA arm during this programme was the highest ever recorded in the UC.

TBAs were acceptable to community (parents) and to the formal health system, as mentioned by vaccinators and the EPI focal person.

#### Facilitating factors for the programme

Coordination between TBAs and vaccinators was exemplary. In coordination with vaccinators, TBAs arranged vaccination camps in the villages. TBAs worked as mobilizers and, on a decided day, they brought children for vaccination. This was possible through organizing a combined meeting between TBAs and vaccinators, which has never been done before in this area.

According to EPI staff, TBAs were particularly important in reducing the dropout rate. TBAs were very helpful in convincing women to strive for vaccine completion. TSVs considered TBAs more effective than vaccinators because culturally being a female, TBAs have access to homes. Being from the local community they can communicate with the mothers easily and they have more information about the new-borns, while vaccinators have less information and access to homes.

Incentive was one of the key motivating factors. This was acknowledged at all levels (by EPI managers), including TBAs, TSVs, DSV and district managers and it seemed to be necessary for the sustainability of this initiative.

#### Challenges encountered

In addition to electricity outages and vaccine unavailability, vaccinators had issues with service tenure. As one vaccinator said:

“Regardless of experience and qualification, we cannot be upgraded from grade 11.”

Vaccinators also considered the coverage area is too big with a shortage of vaccinators due to retirement. Both vaccinators and LHWs considered the polio campaign as hindrance to their work. Because of their frequent visits to households during polio activities, households do not pay much attention to them and parents expect all vaccinations to be done at their doorsteps.

#### Sustainability of the intervention

The TBAs were motivated to work. When asked whether they are willing to work without monetary incentives, a few of them said that they are willing to work without money. One TBA said that although project had concluded now, parents still ask us about new-born vaccinations. We still guide the parents, but are not putting in extra effort to ensure timely vaccination, since we do not have resources to move around the area. EPI staff considered monetary incentives as important factors for TBAs, since TBAs do not have any other source of income.

EPI focal person and DHO considered this program useful. However, they mentioned additional financial resources needed to engage TBAs were not available at the district level.

## DISCUSSION

Although TBAs play a significant role in maternal and child health service delivery in rural and HTR areas in Pakistan, no formal attempts have been made to utilize this workforce for improving EPI coverage. To our knowledge this is the first attempt to generate a referral mechanism between TBAs and vaccinators in Pakistan. Although the TBA intervention area was the most remote in the district and had the lowest coverage, it showed significant improvement over its historical baseline. This pragmatic implementation research increased the enrolment and vaccine completion tremendously in a short period of time. Training of CHWs has shown to improve maternal and child health outcomes (including vaccine coverage) in India and other LMICs [18-24].

Vaccine-related knowledge increased significantly for TBAs after training. Findings were comparable with previous literature [18-20]. Remembering vaccine names was difficult for TBAs, since names were in English. Therefore, TBAs were trained to remember the time interval between vaccine doses (visits) as required by the EPI schedule. A Nigerian study also relied on body memory tools for remembering immunization visits [20]. This strategy proved to be effective since TBAs not only remembered the interval but also referred children for timely vaccination. LHWs had refresher trainings regularly by the government health department, and one training was conducted two months before the project training, so LHWs performed well on pre-tests as well [14,15].

The TBAs and vaccinators had a good liaison. This project aimed to generate a referral mechanism between TBAs and vaccinators where TBAs were sending children to EPI centres. However, in some villages, due to long distances to facilities, families were reluctant to visit fixed centres. Instead, vaccination was done in villages by vaccinators. TBAs collected all eligible children in the village and called the vaccinator on a planned day for vaccination. This arrangement ensured vaccination of children at more convenient sites and reduced efforts of the vaccinator going door-to-door.

The TBAs were enthusiastic and cooperated throughout project. The initiative of involvement of TBAs in vaccination was very welcoming from all stakeholders including vaccinators, EPI managers and parents. A qualitative inquiry conducted in Chitral, Pakistan reported that due to proximity of TBAs with village women, they have a pivotal role in promoting maternal and child health [21].

Polio campaigns were hindering routine immunization activities. For example, LHWs substantial workdays were consumed by polio activities. LHWs’ evaluation reports suggest that, on average, LHWs are spending 150 working days per year for polio related activities [15]. Besides, most households expected vaccinators to visit their homes for vaccination. Previous literature from Pakistan also suggests that due to frequent visits by polio workers during national immunization days (NIDs), people expect vaccinators to visit homes for routine vaccination also [11].

LHWs were covering only 60% of population in selected sub-district, and it was comparable to the national level [14,15]. Extension of formal health care system in the unserved HTR areas would be a costly intervention. With the right training, female CHWs (TBAs and volunteers) can be effective social mobilizers for vaccination. Consideration should be given to providing a monetary incentive to TBAs. Provision of incentive was a major barrier in the continuation of the initiative. Since some CHWs were involved in polio campaigns, they all usually receive incentives. This has created an environment where volunteer work without any incentive was difficult. We attempted to find volunteers without provision of incentives, but failed. Nonetheless, considering the minimal cost (US$8) per complete vaccination of the child, it is worthwhile if we consider the overall cost of the vaccination per child. After including economic and social benefits, return on immunization’s investment is estimated at 44 times the cost of immunization in LMICs [22].

These underserved populations in HTR areas have greatest potential for gains. Therefore, provision of immunization to these populations even at higher cost should be considered cost-effective. However, this cost is comparable with global and regional estimates. According to WHO estimates, average health system cost per child is around US$ 32.6. About 41% of this cost (US$13.36) is related to human resources alone [23]. The calculation made on provincial data from Pakistan, the cost of fully immunizing a child was eestimated at USD$64, and it increases to US$81 in Baluchistan province due to decrease in population density. The HTR areas due to sparse population have greater management challenges which will increase potential cost of immunization [24] Thus, incremental cost accrued during this project was comparatively minimal to expand services in HTR areas. However, a detailed cost-effective or cost-benefit study may be carried out before implementation at scale.

### Challenges and limitations

As a shortcoming to the project we consider that the duration of the project was very short. Although there was an initial improvement in vaccine coverage in the TBA arm, due to the short project time, a steep decline in coverage followed one month later for the BCG vaccine. Therefore, a project with a reasonable extended time period would provide more answers for practical implementation of this strategy.

Due to frequent administrative changes including other initiatives as well as systematic differences in infrastructure which was inherent in study, it was impractical to compare the LHW and TBA areas for improvement in vaccination. During this intervention, the only vaccinator in the LHW arm was transferred. However, another vaccinator from a neighbouring UC was visiting the LHW arm once a week. However, this arrangement may have decreased vaccination coverage in the LHW arm. Thus, we did between group and within group differences to control for unintended extraneous factors. Therefore, we resorted to comparing the overall district-level coverage during the same period with the TBA intervention area. In order to strengthen our analysis, we also compared the previous year’s (2016) vaccination coverage for the district during the same period and the geographical areas for the current year and found significant improvement in enrolment of newborns for vaccination, particularly completion of vaccination (**Table 2**). Nonetheless, the difference and improvement in coverage between TBA arm, LHWs arm and rest of the district was large which cannot be explained by possible extraneous confounders. Since TBAs are not included in the formal health system the research team did not have an available listing of TBAs. The research team had to go village-to-village to identify TBAs. All identified TBAs were covering only 30% of the population. Thus, a mapping of TBAs and similar workers are needed to undertake such initiatives.

## CONCLUSION

About 40% of the Pakistani population living in remote rural areas are not served by LHWs. A TBA-based referral system has potential to improve vaccine enrolment and vaccine completion in these unserved areas. This system is acceptable to population and feasible in HTR areas due to the availability of TBAs. Thus, a policy decision needs to be made to provide coverage for these areas. Consideration should be given to providing a monetary incentive to TBAs. Although, the cost associated with extension of the vaccination coverage to last mile seems minimal. However, a detailed cost-effective or cost-benefit study may be carried out before implementation at scale.
